# PDIP46 (DNA polymerase δ interacting protein 46) is an activating factor for human DNA polymerase δ

**DOI:** 10.18632/oncotarget.7034

**Published:** 2016-01-27

**Authors:** Xiaoxiao Wang, Sufang Zhang, Rong Zheng, Fu Yue, Szu Hua Sharon Lin, Amal A. Rahmeh, Ernest Y. C. Lee, Zhongtao Zhang, Marietta Y. W. T. Lee

**Affiliations:** ^1^ Department of Biochemistry and Molecular Biology, New York Medical College, Valhalla, New York, USA

**Keywords:** DNA polymerase δ, DNA replication, PDIP46, POLDIP3, SKAR

## Abstract

PDIP46 (SKAR, POLDIP3) was discovered through its interaction with the p50 subunit of human DNA polymerase δ (Pol δ). Its functions in DNA replication are unknown. PDIP46 associates with Pol δ in cell extracts both by immunochemical and protein separation methods, as well as by ChIP analyses. PDIP46 also interacts with PCNA via multiple copies of a novel PCNA binding motif, the APIMs (AlkB homologue-2 PCNA-Interacting Motif). Sites for both p50 and PCNA binding were mapped to the N-terminal region containing the APIMs. Functional assays for the effects of PDIP46 on Pol δ activity on singly primed ssM13 DNA templates revealed that it is a novel and potent activator of Pol δ. The effects of PDIP46 on Pol δ in primer extension, strand displacement and synthesis through simple hairpin structures reveal a mechanism where PDIP46 facilitates Pol δ4 synthesis through regions of secondary structure on complex templates. In addition, evidence was obtained that PDIP46 is also capable of exerting its effects by a direct interaction with Pol δ, independent of PCNA. Mutation of the Pol δ and PCNA binding region resulted in a loss of PDIP46 functions. These studies support the view that PDIP46 is a novel accessory protein for Pol δ that is involved in cellular DNA replication. This raises the possibility that altered expression of PDIP46 or its mutation may affect Pol δ functions in vivo, and thereby be a nexus for altered genomic stability.

## INTRODUCTION

Genomic stability in all organisms begins with the process of DNA replication, which is performed by replicative DNA polymerases endowed with exquisite fidelity. An understanding of the properties and regulation of the replicative DNA polymerases is therefore of crucial significance in the context of the maintenance of genome stability. Thus, the mechanisms by which the high fidelity of DNA polymerases is achieved have been extensively studied [1]. In human cells, there are two proofreading DNA polymerases, Pol δ and Pol ε. Mutations that alter the properties of Pol δ and Pol ε are of direct significance as a potential of genomic instability [2, 3]. It is well recognized that defects in the proofreading abilities of DNA polymerases in a broad range of organisms play a crucial role in increased mutation rate and genomic instability [4]. However, it is only recently that increasing information on alterations of Pol δ and Pol ε that are associated with cancer has emerged, as evidenced in studies of colorectal and endometrial cancer [5, 6]. There remains a great deal yet to be learned about mechanisms that could affect the properties of the replicative polymerases and contribute to cancer etiology.

There has been significant progress in the study of human Pol δ and its regulation [7, 8]. The human Pol δ holoenzyme (Pol δ4) is a heterotetramer consisting of p125 (the catalytic subunit), p50, p68 [9], and a fourth subunit, p12 [10], that is not present in *S. cerevisiae* Pol δ [11]. Pol δ4 can be converted to the trimeric form (Pol δ3) by the proteasomal destruction of p12 in response to DNA damage [12, 13]. Pol δ3 is a physiologically active enzyme that is engaged in DNA repair [14]. Pre-steady state kinetic analyses of Pol δ4 and Pol δ3 have shown that the p12 subunit exerts a profound influence on the kinetic constants of Pol δ, such that Pol δ3 exhibits a decreased tendency for lesion bypass, increased stalling at template lesions, and a greater proofreading ability through alteration of the rate constants for the polymerization step (*k*_pol_) and the translocation of the primer terminus from the polymerase to the 3′ to 5′ exonuclease catalytic sites (*k*_pol-exo_) [15, 16]. These studies have shown that the fidelity of Pol δ may be altered through interaction of the p12 subunit with the catalytic core. A balance between Pol δ4 and Pol δ3 appears to be required *in vivo*, since reduced expression of p12 in cancer cells is associated with an increased genomic instability and a poor prognosis for certain lung cancers [17, 18]. The example of p12 also raises a possibility for the existence of other protein interactors of Pol δ that could affect its kinetic properties, including processivity and fidelity.

Pol δ3 is also formed during the normal cell division cycle at the G1/S transition, such that it is the predominant form of Pol δ during S phase [19-21], consistent with its having a role in DNA replication. The degradation of p12 is mediated by the ubiquitin ligase CRL4^Cdt2^ [19, 22] that plays a major role in regulating the G1/S transition [23-25]. We have reconstituted the human Okazaki fragment processing system using both Pol δ3 and Pol δ4 [26, 27]. Both cooperate with Fen1 in removal of blocking primers, so that both appear to be capable of participating in Okazaki fragment processing but operate by different mechanisms for the removal of blocking oligonucleotides [8, 27].

Our laboratory has been interested in the search for Pol δ interacting proteins, in order to identify novel proteins that may be involved in regulating or augmenting Pol δ functions. Screening by the yeast two-hybrid system with the p50 subunit of Pol δ as the bait resulted in identification of two Pol δ binding proteins, named PDIP38 and PDIP46 [28]. PDIP46 was rediscovered as SKAR (S6K1 Aly/REF-like target) [29, 30], a target of ribosomal S6K1 kinase that is downstream of the mTOR and PI3K signaling pathways that regulate cell growth in response to nutritional and mitogenic signals [31]. SKAR has a RNA recognition motif (RRM), an abundant nucleic acid binding domain [32], with homology to that of the Aly/REF RNA binding protein that is involved in posttranscriptional regulatory processes and mRNA export [33]. Hyperphosphorylation and activation of S6K1 leads to its binding to SKAR and the phosphorylation of SKAR; this triggers the recruitment of SKAR/S6K1 to the exon junction complex and increases the translational efficiency of newly spliced mRNA [30]. Partial knockdowns of S6K1 or SKAR result in reduction of cell size, and slowed progression through S phase [29].

Other than the original description of the discovery of PDIP46 (also known as POLDIP3) [28], no information has emerged on the functional effects of PDIP46 on Pol δ. Here we report the first detailed analysis of the effects of PDIP46 on Pol δ function. Our studies show that PDIP46 is associated with Pol δ in a cellular context. Mapping of the interaction sites of PDIP46 with p50 and PCNA show that both sites are located in the N-terminal region, and that PDIP46 interacts with PCNA via APIM motifs (AlkB homologue 2 PCNA-Interacting Motif) [34]. We performed a detailed examination of the effects of PDIP46 on human Pol δ activity using assays that assess its ability for processive synthesis on long stretches of DNA, as well as on model oligonucleotide templates. These studies reveal that PDIP46 has a profound effect on the activity of Pol δ, and support the hypothesis that PDIP46 has a role in cellular DNA replication.

## RESULTS

### Association of PDIP46 with Pol δ

**Figure 1:**
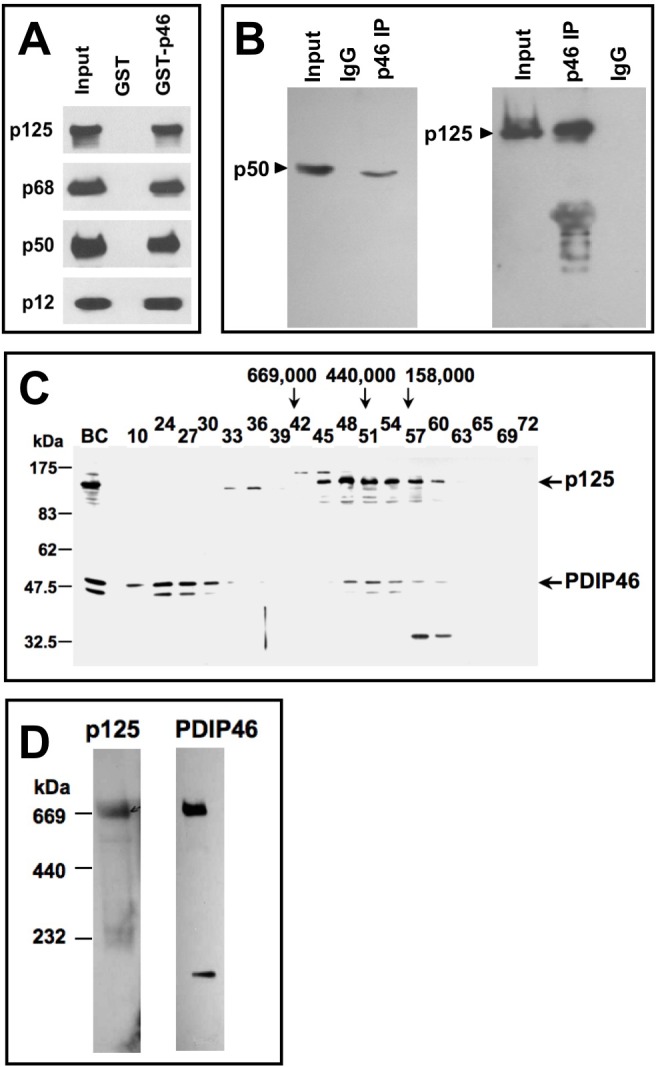
Association of PDIP46 with Pol δ by co-immunopreciptation, gel filtration, and native gradient gel electrophoresis **A**. PDIP46 interacts with the Pol δ4 holoenzyme. GST-PDIP46 (GST-p46) was used to pull-down purified Pol δ. The pull-downs were western blotted for the p125, p68, p50, and p12 subunits of Pol δ. **B**. Co-immunoprecipitation of PDIP46 with Pol δ. HeLa cell extracts were immunoprecipitated with antibodies against PDIP46, and western blotted for the p50 (left panel) and p125 subunits (right panel) of Pol δ. **C**. Nuclear extracts of HEK 293 cells were chromatographed on a Superose 6 FPLC column as previously described [35]. Column fractions were western blotted for p125 and PDIP46. “BC” refers to the nuclear extract. Positions of molecular weight standards are shown on the left. The arrows refer to the elution of protein standards (*M*_r_: thyroglobulin, 669,000; ferritin, 440,000; aldolase, 158,000). **D**. HEK 293 cell lysates were subjected to native (nondenaturing) gradient gel electrophoresis until limiting mobility was reached. Proteins were transferred to nitrocellulose membranes and Western blotted for p125 and PDIP46 (Materials and Methods). The migration positions of marker proteins (thyroglobulin, ferritin and catalase) and their respective native molecular weights are shown on the left.

The only published information on the relationship between PDIP46 and Pol δ was the demonstration of the interaction between PDIP46 and the p50 subunit by the yeast two-hybrid assay [28]. We first determined that PDIP46 interacts with the intact Pol δ heterotetramer, to ascertain that this interaction is not restricted to the free p50 subunit used as the bait in the yeast two-hybrid screen. This was established by the use of GST-PDIP46 pull-down assays, which demonstrated that all four subunits of the Pol δ4 were pulled down (Figure 1A). This interaction was also demonstrated in HeLa cell lysates by immunoprecipitation with polyclonal antibodies against PDIP46, where both the p50 and p125 subunits of Pol δ were pulled down (Figure 1B). The latter result does not necessarily show a direct interaction, since, as will be shown below, PDIP46 also interacts with PCNA. However, a more stringent immunochemical demonstration of the association between PDIP46 was performed, by showing that PDIP46 could be detected in the fractions from the immunoaffinity chromatography of HeLa cell lysates on immobilized p125 antibody; PCNA is not bound to this column (Figure S1, Supplementary Data).

We also determined whether association of PDIP46 with Pol δ could be observed by the formation of higher molecular weight complexes in cultured cell extracts. Nuclear extracts of HEK 293 cells were chromatographed on a Sepharose 6 gel filtration column (Figure 1C). PDIP46 co-eluted in the same fractions as p125, in a region where the ferritin marker (*M*_r_ 440,000) eluted. (Two bands are found for PDIP46 by Western blotting; these bands likely represent the full-length PDIP46 and its smaller alternatively spliced variant [29]). In previous studies, purified recombinant Pol δ4 was found to behave with a molecular weight (232,000-280,000) consistent with its estimated molecular mass [35]. HEK 293 cell lysates were also subjected to nondenaturing gel electrophoresis on gradient gels, under conditions where the gels are run until limiting mobility is reached due to pore size (Figure 1D). We had previously shown that Pol δ in cultured cell extracts migrates at a position similar to thyroglobulin (M_r_ 669,000). As seen in Figure 1D, both the p125 subunit of Pol δ and PDIP46 co-migrate at a position similar to thyroglobulin (*M*_r_ 669,000). Together, these experiments support the idea that PDIP46 associates with Pol δ in a cellular context.

### Association of PDIP46 with Pol δ on chromatin as determined by ChIP analysis

**Figure 2:**
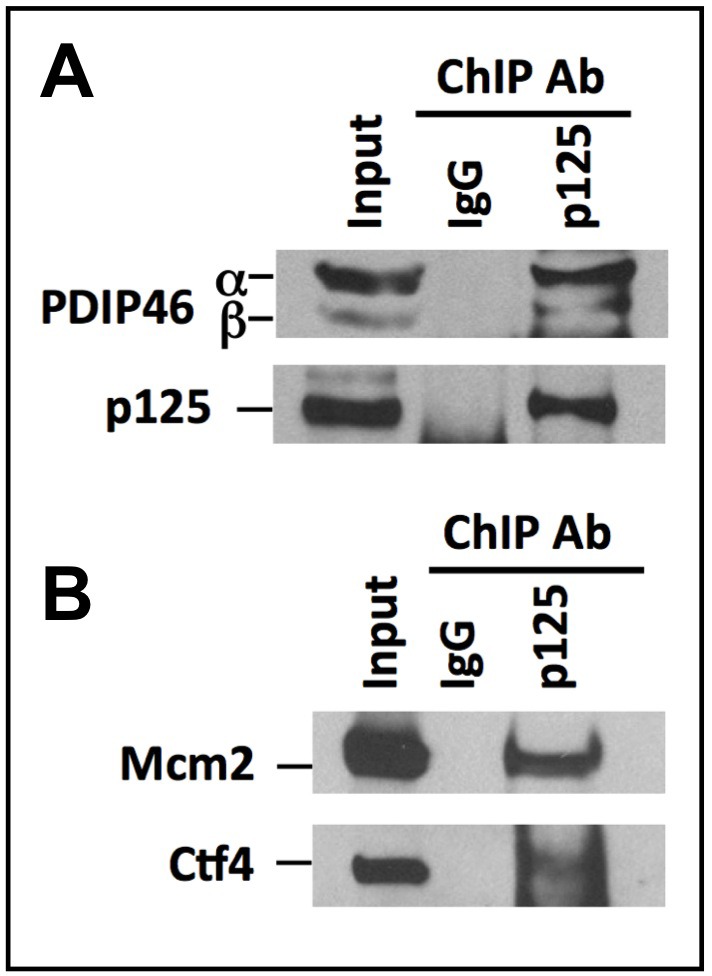
PDIP46 is associated with chromatin bound Pol δ by ChIP analysis using antibody against p125 ChIP analysis was performed with A549 cells as described in “Materials and Methods”. **A**. The immunoprecipitates were western blotted with antibodies against PDIP46 and the p125 subunit of Pol δ. IgG refers to the control immunoprecipitation with non-immune serum. The bands marked “α” and “β” refer to the full-length PDIP46 and its minor spliced variant, respectively. **B**. ChIP analysis was performed as in (A) and western blotted for MCM2 and Ctf4 which were used as positive controls.

An important step for establishing a role of PDIP46 in DNA replication is the determination of whether it is bound to chromatin in association with Pol δ and other replication proteins. We performed chromatin immunoprecipitation of HeLa cells, using an antibody against p125 [14], and immunoblotted for PDIP46. PDIP46 was co-immunoprecipitated with p125 by ChIP analysis (Figure 2A). As a positive control, we performed the same ChIP analysis and blotted for two proteins associated with the replication fork, Mcm2 and Ctf4 (Figure 2B). Mcm2 is a subunit of the Mcm2-7 helicase that together with Cdc45 and the GINS complex form the CMG replicative helicase in yeast [36] and humans [37,38]. Ctf4 (chromosome transmission fidelity 4) is important for sister chromatid cohesion and DNA replication; it associates with the CMG helicase and interacts with the replicative polymerases, most strongly to Pol α, pointing to a role in replisome assembly [39, 40]. Taken together with data of Figure 1, these results provide evidence that PDIP46 is associated with Pol δ at the replication fork on chromatin.

### Mapping of the interaction domains between p50 and PDIP46

**Figure 3:**
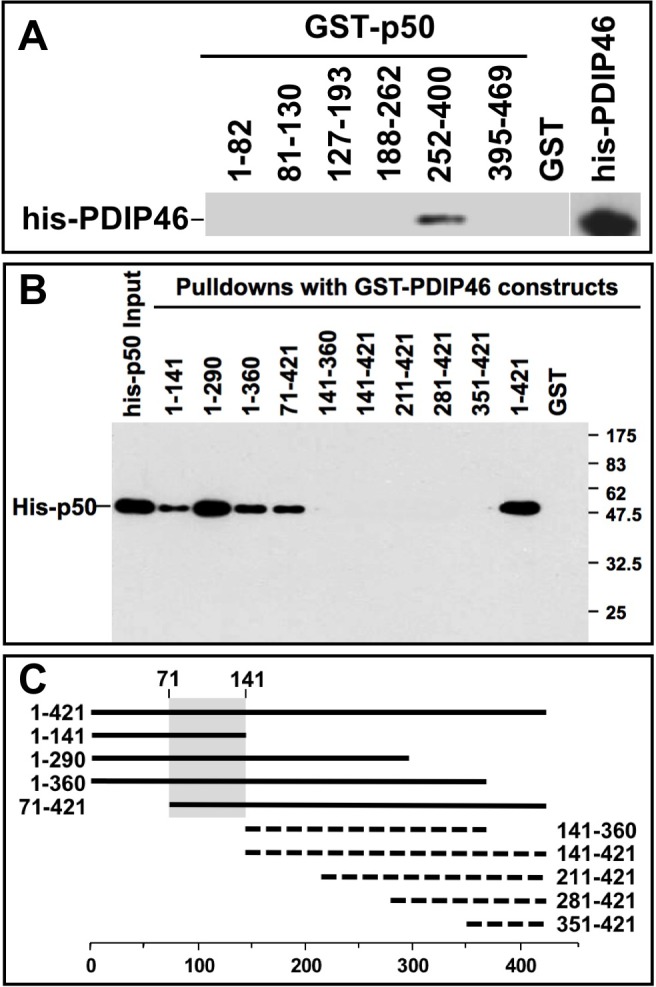
Mapping of the interaction sites between p50 and PDIP46 **A**. Mapping of the region of p50 that interacts with PDIP46. GST-fusion constructs of p50 were used to pull-down his-PDIP46, and western blotted for PDIP46. Only the deletion mutant containing residues 252-400 of p50 interacted with PDIP46. **B**. Mapping of the PDIP46 region that interacts with the p50 subunit of Pol δ. GST-PDIP46 deletions were used for pull-down assays of his-p50, and western blotted for p50. **C**. Diagrammatic summary of the data of panel B. Solid bars show those deletion constructs that interacted with p50, and the dashed lines show those that did not. The shaded area shows the common region of the PDIP46 deletion mutants that interacted with p50 (residues 71-141).

The region of p50 involved in PDIP46 interaction was mapped using pull-down assays by the use of GST-p50 deletion constructs to pull down his-tagged PDIP46 (Figure 3A). This was shown to be between residues 252-400 of p50. The interaction domain of PDIP46 for binding to p50 was also mapped by the use of GST-fusion deletion constructs of PDIP46 to pull down his-p50 (Figure 3B). A diagram of the mutants that pulled down his-p50 showed that the binding region on PDIP46 for p50 lies between residues 71-141 in the N-terminus (Figure 3C, shaded region).

### PDIP46 interacts with PCNA: identification of PDIP46 as a novel member of the group of proteins that interact with PCNA via APIM motifs

**Figure 4:**
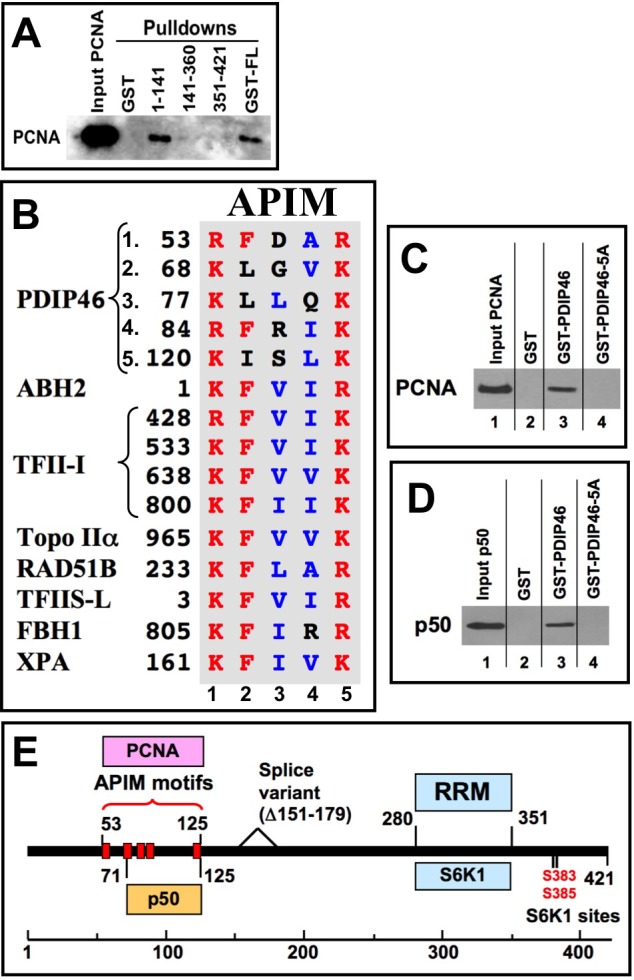
PDIP46 interacts with PCNA, and does so via APIM motifs **A**. Mapping of the PDIP46 domain that interacts with PCNA. GST-PDIP46 deletion mutants were used to pull-down PCNA, and western blotted for PCNA. Only the GST-1-141 fusion protein interacted with PCNA. **B**. The N-terminus of PDIP46 harbors 5 APIM motifs. The alignment shows the five APIM motifs of PDIP46, together with those of the oxidative demethylase ABH2, the four APIMs of TFII-I, Topo IIα, Rad51B and TFIIS-L [34], FBH1 (F-box helicase) [44] and XPA [43]. Residues in red show the conserved basic residues at positions 1 and 5, as well as the phenylalanine at position 2, while those in blue are the aliphatic residues at positions 3 and 4. All sequences shown are those of human proteins. **C**. Mutation of the APIMs of PDIP46 leads to loss of PCNA binding. The conserved residues in positions 1, 2 and 5 of the APIM motifs were mutated to alanines (PDIP46-5A). GST-PDIP46 and the GST-PDIP46-5A mutant were used in pull-down assays of PCNA. **D**. Mutation of the APIMs of PDIP46 also leads to loss of p50 binding. GST-PDIP46 and the GST-PDIP46-5A mutant were used in pull-down assays of his-p50. **E**. Domain map of PDIP46 showing the location of binding regions (boxed) for p50, PCNA (APIM motifs), RRM, and S6K1. The two S6K1 phosphorylation sites and the region deleted in a minor splice variant [29] are also shown.

We found that PDIP46 also interacts with PCNA. Three GST-PDIP46 fusion constructs were used to perform pull-down assays of PCNA. Only the full-length PDIP46 and the N-terminal fragment (residues 1-141) were able to pull-down PCNA (Figure 4A). PCNA-binding partners generally possess a PIP-box, a short protein motif that binds to a hydrophobic pocket on PCNA [41,42]. However, inspection of this N-terminal region (or the entire PDIP46 sequence) did not reveal any sequences corresponding to a canonical PIP-box. The N-terminal sequence harbors five repeats of a short sequence (Figure 4B) which we identified as members of an alternative PCNA binding motif [34], the APIM (AlkB homologue 2 PCNA-Interacting Motif). The APIM consists of five residues with the consensus sequence [KR]-[FYW]-[LIVA]-[LIVA]-[KR] and was initially identified as a novel PCNA binding motif in human oxidative demethylase ABH2 (AlkB homologue 2), a DNA repair enzyme [34]. Bioinformatics searches identified over 200 human proteins that contain APIMs; these include many proteins that are involved in genomic maintenance (DNA repair, DNA replication and cell cycle control) [34]. However, functional PCNA binding by APIMs has been demonstrated in only seven proteins to date - ABH2, the transcription factor TFII-I, Topo IIα, Rad51B, TFIIS-L [34], the nucleotide excision repair protein XPA [43], and the F-box helicase, FBH1, that is involved in homologous recombination [44]. The alignment of the APIMs of PDIP46 with those of these seven proteins show that they conform to the motif, with the exceptions of the conservative replacements of phenylalanine with leucine or isoleucine in three of the motifs (APIMs 2,3,5), and all have additional variations in the aliphatic residues of positions 3 or 4 (Figure 4B). A BLAST (tblastn) search showed that the region (residues 50-130) containing all five PDIP46 APIMs is almost completely conserved in mammalian species, while the full-length sequence is >90% conserved. PDIP46, while highly conserved in higher vertebrates, does not appear to be present in lower vertebrates [29].

In order to demonstrate that the APIMs are functionally responsible for the binding of PCNA by PDIP46, we took the approach of mutating all five of the motifs to generate the PDIP46-5A mutant to avoid the complexity that might arise from the ability of the individual motifs to bind PCNA. Residues at positions 1 (R/K), 2 (F/L/I) and 5 (R/K) in all five of the APIMs (Figure 4B) were mutated to alanines. GST-PDIP46 and GST-PDIP46-5A were used to pull-down PCNA (Figure 4C). PDIP46-5A exhibited near complete loss of PCNA binding. These results eliminate the involvement of a variant PIP-box elsewhere in PDIP46, noting that Topo IIα [34] and FBH1 [34] possess both APIMs and PIP-boxes. More detailed mutational analyses will be needed to determine which of the five APIMs of PDIP46 are needed for the interaction with PCNA. However, it is noted that the first four APIMs are tightly clustered, with the spacing being 10, 4, 2 and 32 amino acid residues between the five APIMs so that there may be spatial or conformational constraints on which of these interact with PCNA.

These data narrow down the region involved in PCNA binding to the region containing the five APIMs (residues 53 to 125). This closely overlaps the region of PDIP46 for p50 binding (residues 71-141) that was mapped by deletion mutagenesis (Figure 3C). The possibility that mutation of the APIMs might also affect p50 binding was considered. This was indeed found to be the case, as the PDIP46-5A was found to have lost the ability to bind to p50 (Figure 4D). This allows the delimitation of the p50 binding region from residues 71-141 (Figure 3B, 3C) to residues 71-125. Thus, the PDIP46-5A mutant is one that has lost interaction with both PCNA and Pol δ.

The location of the APIMs, PCNA and Pol δ binding regions are shown diagrammatically in the domain map of PDIP46 (Figure 4E). Both the p50 and PCNA binding regions of PDIP46 are located in the N-terminus, while the binding region for S6K1, which is important for its functions as the SKAR protein, are in the C-terminus within the RRM. The separation of the interaction sites for p50 and PCNA and the RRM are significant as they are consistent with the idea that PDIP46/SKAR is a bifunctional protein.

### PDIP46 is a potent activator of Pol δ4 in primer extension assays which require highly processive synthesis

We had examined the effects of PDIP46 on Pol δ using poly(dA)_4000_/oligo(dT)_50_ as the substrate. This assay is commonly used for the assay of Pol δ activity. In this assay Pol δ was inhibited with half maximal inhibition at about 1 μM of PDIP46. The inhibition could be relieved by increasing the concentration of PCNA (Figure S2, Supplementary Data), indicating a competition between Pol δ and PDIP46 for PCNA. Such effects are likely to be non-physiological, given the concentration levels needed, and are not unexpected as they could be observed with any other PCNA binding protein. In previous studies of PDIP38, which also binds PCNA, we observed that it inhibited the activity of Pol δ when assayed using poly(dA)_4000_/oligo(dT)_50_ as the substrate [45]. These effects occurred at micromolar levels of PDIP38, and are likely due to competition with Pol δ for PCNA.

**Figure 5:**
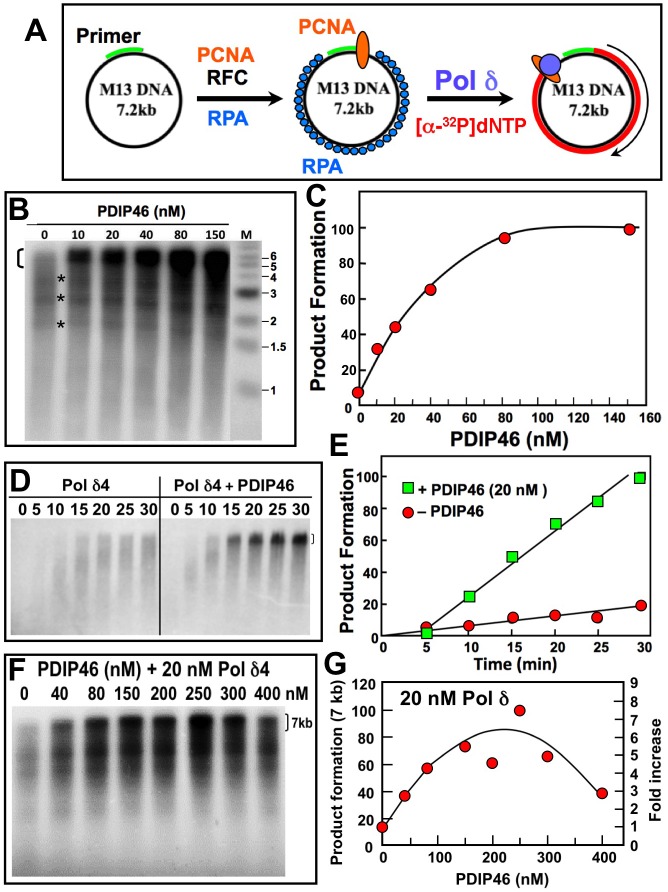
PDIP46 stimulates product formation by Pol δ4 in the M13 assay **A**. Diagram of the M13 assay of Pol δ activity. Singly primed M13 ssDNA (left) is loaded with PCNA with RFC, and RPA single stranded DNA binding protein (center); Pol δ4 and [α-^32^P]-dATP is added to extend the primer up to the full-length product (right). **B**. Effects of increasing concentrations of PDIP46 on Pol δ4 activity. Pol δ4 concentration was 5 nM, M13 ssDNA was 2.5 nM, and PCNA was 6 nM (Materials and Methods). Reactions were incubated at 37° C for 25 min. Products were analyzed by electrophoresis on 1.2% alkaline agarose gels and were visualized by phosphorimaging. Lane M shows the migration of the markers. The bracket on the top left indicates the region that was used for quantitation of full-length 7 kb products. The asterisks show bands where pausing of the reactions occurred. **C**. Full-length product formation for panel B was quantified, and plotted as relative product formation against PDIP46 concentration. The data were fitted to a one site binding hyperbola using Prism software, and gave an apparent *K*_D_ of 34 ± 7.7 nM (R^2^ = 0.98). **D**. Time dependence of product formation by Pol δ4 in the M13 assay in the presence of PDIP46. Pol δ4 (10 nM) was assayed on singly primed M13 in the absence (left panel) and presence (right panel) of PDIP46 (20 nM); the reactions were analyzed after 5, 10, 15, 20, 25 and 30 min. **E**. Product formation of the full-length products in panel D was quantified, and plotted as relative product formation against time. Data in the absence of PDIP46 are shown as circles, and those in the presence of 20 nM PDIP46 are shown as squares. **F**. The effects of higher concentrations (0-400 nM) of PDIP46 on Pol δ (20 nM) assayed on the M13 substrate. Reaction times were 15 min. **G**. The full-length products for panel F were quantified and plotted against PDIP46 concentration.

We re-assessed the effects of PDIP46 using singly primed M13 DNA as the substrate (Figure 5A). The single stranded M13 DNA is ca. 7 kb in size, and presents a more complex template than the homopolymeric poly(A)_4000_ used in the poly(dA)/oligo(dT) assay, as it contains regions of secondary structure. This assay has been used to examine the ability of Pol δ/PCNA to perform processive synthesis to the full-length products of ca. 7 kb [46], and is regarded as an quasi-reconstitution assay that provides an *in vitro* assessment of Pol δ capability in leading strand synthesis in a processive manner [35, 38, 47, 48]. PCNA is first loaded onto the primed M13 DNA by its clamp loader, RFC, in the presence of RPA and ATP (Figure 5A). Pol δ activity in this assay is dependent on the addition of RPA single stranded binding protein [46].

PDIP46 was found to be a potent stimulator of formation of the full-length 7 kb product by Pol δ4. The formation of products at or near full-length extension of the primer is dramatically increased by PDIP46 within a concentration range from 0-150 nM (Figure 5B). Analysis of product formation in the 7 kb range showed that the reactions displayed saturation kinetics with increasing concentration of PDIP46, and at the highest concentration used, this amounted to a *>*10 fold increase in product formation (Figure 5C). The data were fitted to a one site binding hyperbola, giving an apparent *K*_D_ of 34 ± 7.7 nM (R^2^ = 0.98) (Figure 5C).

The time course of the formation of full-length M13 DNA in the presence of 20 nM PDIP46 is shown in Figure 5D. Even at 20 nM, PDIP46 dramatically accelerates the formation of the full-length M13 products by Pol δ4 (Figure 5D, 5E).

With regard to our previous observations of an inhibitory effect of PDIP46 in the poly(dA)/oligo(dT) assay, we could also observe inhibition when we used high concentrations of PDIP46 (Figure 5F, 5G). We re-examined the effects of PDIP46 on Pol δ activity in the poly(dA)/oligo(dT) assay using a lower range of concentrations, but were unable to detect any stimulation of Pol δ activity (data not shown). It is noted that the template in this case is a homopolymer, and lacks any sequence complexity.

The effects of increasing Pol δ4 levels in the absence and presence of a fixed PDIP46 concentration (100 nM) were examined (Figure S3A, S3B, Supplementary Data). Product formation was quantitated for the major products in the 3-7 kb range as well as for the 7 kb range (Figure S3C, D, Supplementary Data). In both cases, apparent saturation of the product formation was observed, that was increased in the presence of PDIP46. This result is consistent with the possibility that there may be an effect on the intrinsic activity of Pol δ.

Overall, our findings show that PDIP46 exhibits a remarkable ability to stimulate product formation by Pol δ4, by as much as an order of magnitude. While human Pol δ4 is capable of processive synthesis on M13 ssDNA templates in the presence of PCNA, it has been reported to dissociate frequently [38,49], and differs from yeast Pol δ which is highly processive [48], i.e., its processivity is not such that it can synthesize the entire M13 DNA in a single binding event. This is evident in that M13 DNA possesses regions of secondary structure that give rise to observable pause sites (Figure 5B, asterisks). These pause sites represent the slowing of Pol δ synthesis through these regions. Facilitation of Pol δ synthesis through these regions could contribute to an increase in apparent processivity of Pol δ. The stimulation of product formation by PDIP46 could be caused by contributions of several mechanisms that include direct activation of Pol δ, an actual effect on processivity, or the ability to facilitate Pol δ elongation through regions of secondary structure. Further complexities involved in understanding how PDIP46 affects Pol δ activity arise because it binds to both Pol δ and with PCNA and has the potential to act as a bridge to stabilize their interaction on DNA.

### PDIP46 stimulates primer extension by Pol δ on model oligonucleotide substrates

**Figure 6:**
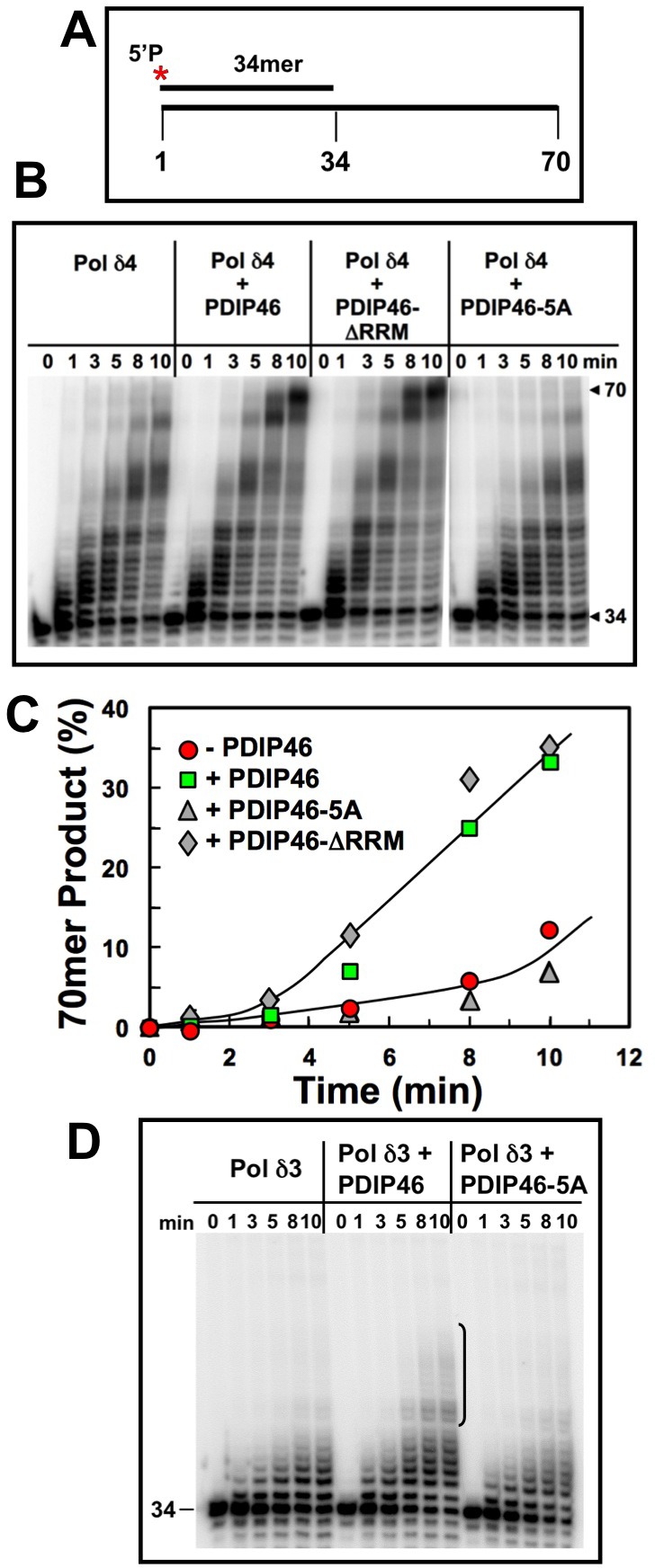
PDIP46 and PDIP46-ΔRRM but not PDIP46-5A stimulate primer extension by Pol δ4 on oligonucleotide substrates in the absence of PCNA **A**. Oligonucleotide substrate for primer extension. A 5′-[^32^P]end-labeled 34mer primer was annealed to a 70mer template. The asterisk denotes the labeling. **B**. Effects of PDIP46 and PDIP46-5A (50 nM) on primer extension by Pol δ4 in the absence of PCNA. The concentration of reactants were DNA substrate (100 nM), Pol δ4 or Pol δ3 (5 nM), PDIP46, PDIP46-ΔRRM or PDIP46-5A (50 nM). The reactions were performed for times ranging from 0-10 min. Reaction products were resolved by electrophoresis on sequencing gels and visualized by phosphorimaging (Materials and Methods.). **C**. The full-length 70mer primer extension products for panel B were quantified and plotted (as % of primer converted to 70mer) against time. Data for Pol δ4 in the absence of PDIP46 is shown as solid circles, with PDIP46 as solid squares, with PDIP46-ΔRRM as shaded diamonds and with PDIP46-5A as shaded triangles. **D**. Effects of PDIP46 and PDIP46-5A on Pol δ3 activity in the absence of PCNA. Reactions were performed as described in B. The vertical bracket on the gel show a region of primer extension that is increased in the presence of PDIP46.

In order to further explore the effects of PDIP46 on Pol δ4 activity and to gain insights on its potential mode of action, we examined its effects using oligonucleotide substrates, identical to those we had previously utilized for the reconstitution of human Okazaki fragment processing [26, 27]. The component reactions include primer extension as well as strand displacement reactions. The oligonucleotide substrate consisted of a 5′ end-labeled 34mer annealed to a 70mer template (Figure 6A). We also tested the PDIP46-5A and PDIP46-ΔRRM mutants to establish that the functional effects resided in the binding regions for Pol δ and PCNA. The PDIP46-ΔRRM mutant is one in which the RRM domain (residues 280-351) in the C-terminus were deleted. Here, we were concerned that the RRM motif, which binds RNA, is also able to bind ssDNA, and could have a potential effect on Pol δ.

The effects of PDIP46 on Pol δ4 were first examined in the absence of PCNA. Pol δ4 produced a ladder of products, as expected from a distributive mode of synthesis. PDIP46 strongly increased formation of full-length 70mer extension products (Figure 6B, 6C). Removal of the RRM did not affect the ability of PDIP46 to stimulate Pol δ4 activity. Mutation of the APIMs abolished the effects of PDIP46, showing that the functional effects are due to Pol δ interaction. The ability of PDIP46 to activate Pol δ4 in the absence of PCNA provides clear evidence that there is a direct effect on Pol δ activity, independent of the presence of PCNA. While the action of Pol δ in the context of its functions in replication require its interaction with PCNA, these findings are highly significant in the context of understanding the mechanism of the effects of PDIP46 on Pol δ. This is because the properties of Pol δ are dictated by a complex kinetic scheme [7, 16] shared by other replicative DNA polymerases [1, 50-52]. Alterations in these kinetic constants can not only alter steady state activity, but also fidelity and processivity [50].

We examined the effects of PDIP46 on Pol δ3 (Figure 6D). In the absence of PCNA, Pol δ3 activity is lower than that of Pol δ4, and is only weakly stimulated by PDIP46. This effect is abolished in the PDIP46-5A mutant, confirming that the effects are due to PDIP46.

**Figure 7:**
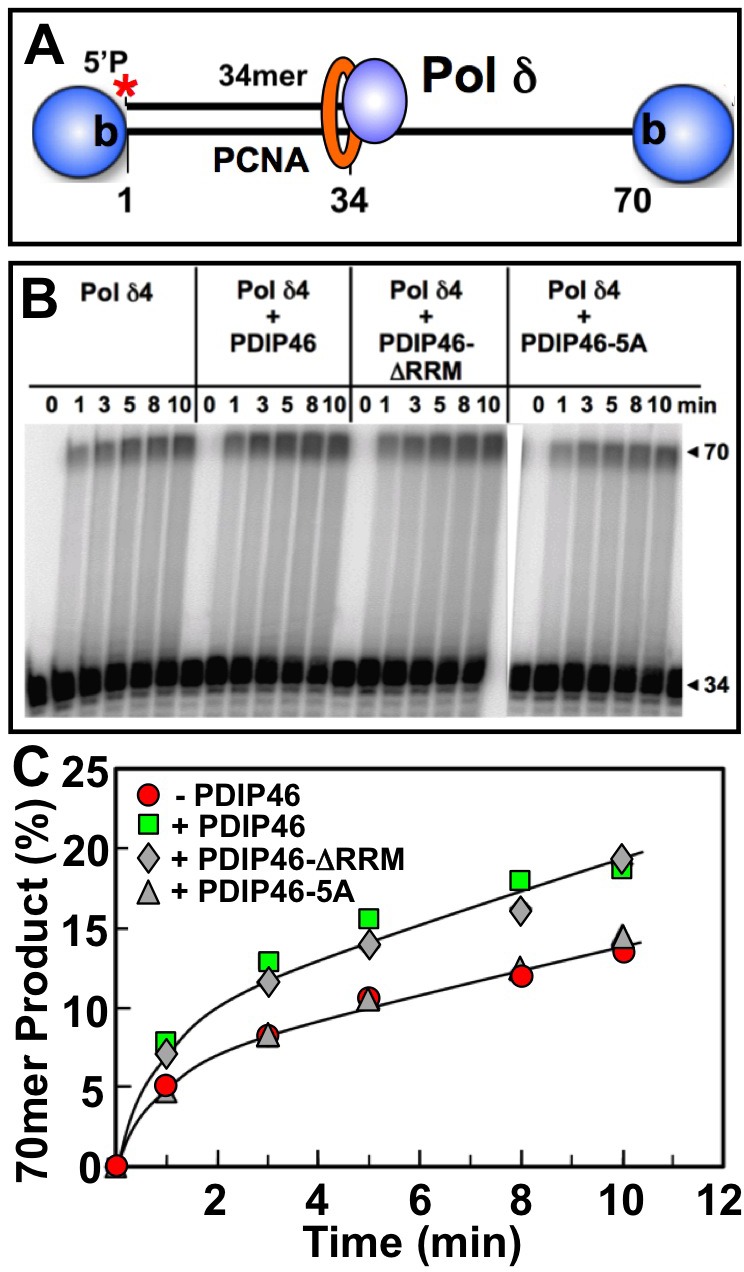
Effects of PDIP46, PDIP46-ΔRRM and PDIP46-5A on primer extension by Pol δ on oligonucleotide substrates in the presence of PCNA **A.** Oligonucleotide substrate for primer extension. A 5′-[^32^P]end-labeled 34mer primer was annealed to a 70mer template, and was then blocked with streptavidin (shaded sphere). PCNA was then loaded onto the substrate with RFC. **B**. Effects of PDIP46 and PDIP46-5A (50 nM) on primer extension by Pol δ4 in the presence of PCNA. The concentrations of reactants were DNA (50 nM), Pol δ4 (10 nM), PCNA (50 nM), PDIP46 or its mutants (50 nM). Conditions used were as described in Materials and Methods. The reactions were performed for times ranging from 0-10 min. Reaction products were resolved by electrophoresis on sequencing gels and visualized by phosphorimaging. **C**. Amounts of 70mer formed in panel B were determined and plotted against time. Data for Pol δ4 in the absence of PDIP46 is shown as solid circles, with PDIP46 as solid squares, with PDIP46-ΔRRM as shaded diamonds and with the PDIP46-5A mutant as shaded triangles.

The effects of PDIP46 on Pol δ4 in the presence of PCNA were examined. For these experiments the same substrate was used, but the biotinylated template ends were blocked with streptavidin (Figure 7A) and PCNA was loaded with RFC [27]. Primer extension by Pol δ4 was markedly stimulated by PCNA alone, as expected, with the shift to processive synthesis seen by the appearance of the full-length extension products even at the earliest time points (Figure 7B, left panel). PDIP46 nevertheless further stimulated Pol δ4 as shown by quantitation of the 70mer full-length products. As with the primer extension experiments in the absence of PCNA (Figure 6B), no effects of the deletion of the RRM domain were seen, while the PDIP46-5A lost the ability to stimulate Pol δ4 (Figure 7B, 7C).

The smaller stimulation by PDIP46 in the presence of PCNA compared to those in the absence of PCNA may be due to the overriding effects of PCNA. (We have also used the unblocked template, and have obtained essentially similar results, consistent to our previous observations [27]. These findings also eliminate the possibility that PDIP46 affects the loading of PCNA by RFC.)

### Effects of PDIP46 on strand displacement by Pol δ on a model oligonucleotide substrate

**Figure 8:**
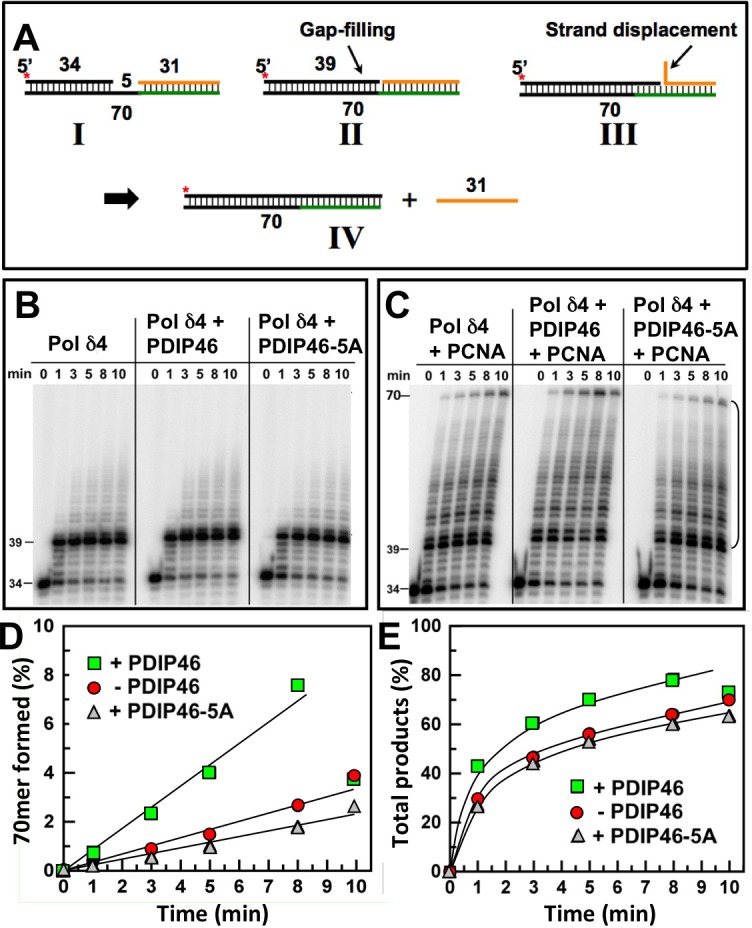
PDIP46 stimulates strand displacement by Pol δ4 **A**. Oligonucleotide substrate for primer strand displacement assays. A 5′-[^32^P]end labeled 34mer primer was annealed to a 70mer template as in Figure 6, together with a downstream blocking 31mer to leave a 5nt gap. The asterisk denotes the labeling. The concentration of reactants were DNA template (100 nM), Pol δ4 (5 nM), PDIP46 or PDIP46-5A (50 nM), and PCNA (100 nM) when added. Reactions were performed for the indicated times. **B**. Effects of PDIP46 and PDIP46-5A (50 nM) on strand displacement by Pol δ4 in the absence of PCNA. Reaction products were visualized by phosphorimaging. **C**. Effects of PDIP46 and PDIP46-5A on strand displacement by Pol δ4 in the presence of PCNA. Reaction products were visualized by phosphorimaging. **D**. The 70mer full-length primer extension products for C, reflecting complete strand displacement of the blocking 31mer oligonucleotide, were quantified and plotted as 70mer formed as % of primer against time. Data points in the absence of PDIP46 are shown as solid circles, with PDIP46 as solid squares, and with PDIP46-5A as shaded triangles. **E**. The overall strand displacement products as reflected by primer extension products from the 40mer-70mer (indicated by the bracket in C) were quantified and plotted against time. Data points were labeled as for panel D.

The effects of PDIP46 on strand displacement by Pol δ4 were examined using a 70mer oligonucleotide template with a 5′-[^32^P]end-labeled 34mer primer and a 31mer blocking sequence (Figure 8A) [27]. On this substrate, Pol δ rapidly extends the 34mer primer to fill in the 5nt gap to form a 39mer and then stalls on encountering the 5′ end of the blocking oligonucleotide. Further primer extension then requires displacement of the blocking oligonucleotide, which takes place at a slower rate, and provides an assay for strand displacement. Negligible strand displacement occurred in the absence of PCNA (Figure 8B), consistent with our previous observations that strand displacement by Pol δ4 is dependent on the presence of PCNA [27]. PCNA markedly stimulated strand displacement, giving rise to a ladder of intermediate products and the formation of the 70mer (Figure 8C, left panel). Quantitation of the 70mer strand displacement product or of the combined strand displacement products between the 40-70mer (Figure 8D, 8E) shows quite clearly that strand displacement is stimulated by PDIP46. These effects were lost when the PDIP46-5A was used. Pol δ3 itself has no significant strand displacement activity [27] and was not significantly affected by PDIP46 in this assay (data not shown).

### PDIP46 enhances the ability of Pol δ4 to synthesize through a model oligonucleotide substrate with a hairpin secondary structure

**Figure 9:**
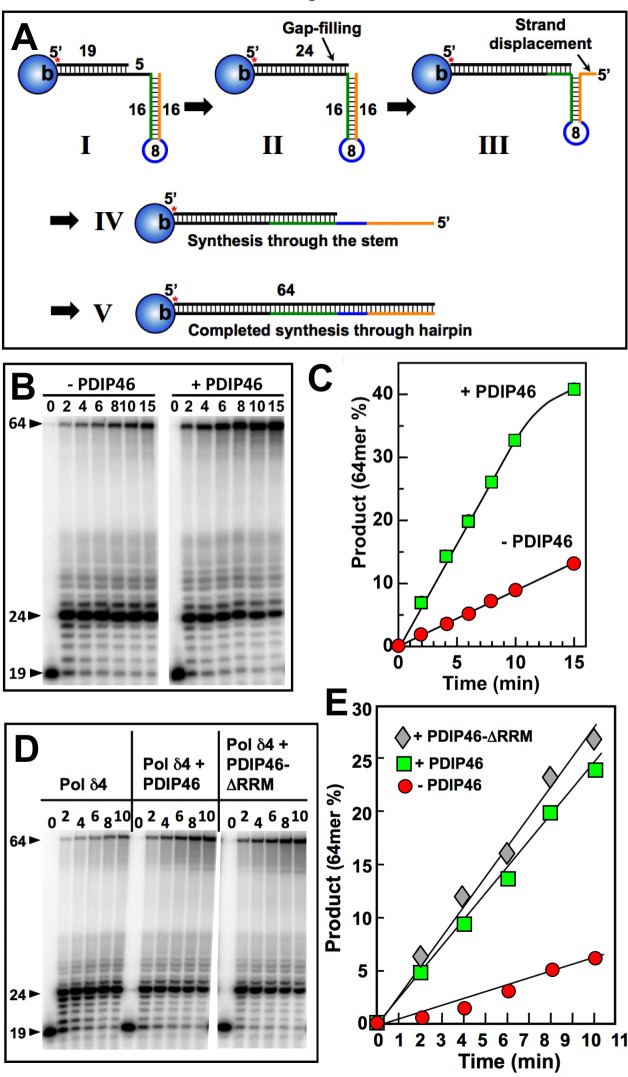
PDIP46 stimulates primer extension by Pol δ4 through a template with a hairpin **A** Diagram of the substrate and expected progression through the stem loop structure. For details see text. **B**. Pol δ4 (15 nM) was reacted for the indicated times with the substrate (50 nM) in the absence and presence of PDIP46 (50 nM) after the loading of PCNA with RFC (Experimental Procedures). The products were analyzed by 8M urea denaturing polyacrylamide gel electrophoresis and visualized by phosphorimaging. The arrowheads indicate the positions of the primer (19nt), the position at the point of primer extension to the 5′-end of the template at the start of the hairpin (24nt), and the full-length product (64nt). **C**. The amounts of 64mer representing synthesis through the hairpin for the phosphorimage in B were quantified. Data are plotted as percentage of primer converted in the absence (solid circles) and presence (solid squares) of PDIP46. **D**. The RRM region is not required for PDIP46 stimulation of Pol δ4. The effects of PDIP46 and the PDIP46-ΔRRM mutant were examined. The concentrations of the reactants were DNA (50 nM), Pol δ4 (10 nM), and PDIP46 or PDIP46-ΔRRM (50 nM). **E**. Formation of the 64mer for panel D was quantified and plotted against time. Data for the control are shown as solid circles, for PDIP46 as solid squares, and for PDIP46-ΔRRM as solid diamonds.

The ability of PDIP46 to stimulate strand displacement activity by Pol δ4 provides insights into its potential mode of action when considering the effects on Pol δ4 function in the context of complex templates such as M13 ssDNA template, viz., that PDIP46 might facilitate synthesis by Pol δ through regions of secondary structure that involve simple stem-loop or hairpin structures. The model substrate consisted of a 64mer template, with a 3′-biotin tag (Figure 9A, “I”). The 5′-end of the template contained a complementary region of 16 nt to form a short stem, and followed by 8 non-complementary nts to form a loop. To this was annealed a 5′-[^32^P] labeled primer, leaving a gap of 5 nt. Streptavidin was used to cap the 3′-biotinylated template end to prevent PCNA from sliding off after it was loaded with RFC [27]. The expected progress of the reaction is shown in a stepwise manner in Figure 9A, to illuminate the analogy with the strand displacement reactions of Pol δ [27]. Pol δ is expected to rapidly extend the primer to fill in the gap until 5′ end of the stem is reached (Figure 9A, “II”). The further process of synthesis through the stem is analogous to strand displacement, noticing that in the process a flap is formed, as the stem region is shortened, giving rise to a familiar stem-loop arrangement (Figure 9A, “III”). Once Pol δ has traversed the stem region (Figure 9A, “IV”) the hairpin is opened and Pol δ is expected to rapidly complete synthesis to the end of the template (Figure 9A, “V”).

The results of the experiment show that Pol δ4 performs synthesis through the hairpin as predicted as can be seen from the gel (Figure 9B). The gap filling is rapid, so that the 19mer is largely converted to the 24mer within the first time point. This is followed by the formation of a ladder of products arising from a slower progression in a process of strand displacement, and a more rapid reaction once the stem region is passed as seen by the absence of intermediates. Quantitation of the phosphorimage showed that PDIP46 (50 nM) readily stimulated the rate of Pol δ synthesis through the hairpin, by a factor of ca. 3.6 fold (Figure 9C). [This is also evident from the disappearance of the 24mer in the presence of PDIP46 (Figure 9B)]. The effects of higher concentrations of PDIP46 were examined. Here, as with the M13 substrate, PDIP46 was inhibitory at high concentrations (Figure S4, Supplementary Data).

We examined the behavior of the PDIP46-ΔRRM mutant (Figure 9D, 9E). The PDIP46 stimulation of Pol δ4 on the hairpin substrate was found to be independent of the RRM motif. The ability of PDIP46 to stimulate strand displacement and synthesis through hairpin structures may underlie its effects on Pol δ4 synthesis in the M13 assay. We also performed a similar experiment with Pol δ3 (Figure S5, Supplementary Data). The rate of 64mer product formation by Pol δ3 was very minimal, about 2% that of Pol δ4. Nevertheless, PDIP46 increased this by ca. 4-fold. An increase in activity with the PDIP46-ΔRRM was observed but this may not be significant due to the low levels of activity.

It is noted that the model of the reactions (Figure 9A) illustrates that passage through the stem region is a reaction that bears resemblance to strand displacement. Comparison of the rates of synthesis in strand displacement (Figure 8D), in which the Pol δ4 concentration was half that used in Figure 9E, shows that the rates of synthesis are roughly comparable in terms of full length products formed.

These experiments suggest a hypothesis whereby the ability of PDIP46 to stimulate Pol δ4 synthesis of the full length M13 substrate can be explained by the cumulative effect of the facilitation of Pol δ4 synthesis through multiple regions of secondary structures. This is presented in more detail in the Discussion.

## DISCUSSION

### Characterization of PDIP46 interactions with Pol δ and PCNA

The studies reported here provide the first detailed examination of the interaction of PDIP46 with Pol δ and PCNA and of its functional effects on Pol δ activity. We established that PDIP46 is associated with Pol δ in cellular extracts by classical protein separation and immunochemical procedures (Figure 1) and that PDIP46 is associated with Pol δ by ChIP analysis with anti-p125 antibody, providing direct evidence that PDIP46 is chromatin bound in the spatial proximity of Pol δ (Figure 2). These observations support the hypothesis that PDIP46 interaction with Pol δ is functionally meaningful in terms of a role in DNA replication.

Detailed analysis of the interaction sites of PDIP46 for the p50 subunit of Pol δ and PCNA, established that these located in the N-terminus (Figures 3, 4). PDIP46 was shown to be a novel member of a group of proteins that interact with PCNA via APIM motifs [34]. Studies using mutants of PCNA in the PIP-box domain and FRET analyses have provided some evidence that the APIMs may bind to the same hydrophobic pocket of PCNA as the PIP-box [53], so that competition between PCNA binding proteins that utilize a PIP-box may occur. However, the biochemical or structural basis of the interaction of the APIM motif with PCNA has yet to be determined. Further studies are required to determine which of the PDIP46 APIM motifs are functionally involved in PCNA binding.

Mutation of all five APIMs leads to loss of both p50 and PCNA binding. This established that the functional effects of PDIP46 are dependent on p50 and PCNA binding (Figure 4). Studies of the PDIP46-ΔRRM mutant show that the effects of PDIP46 on Pol δ4 are independent of the RRM domain associated with the functions of PDIP46 studied as the SKAR protein (Figures 6, 7). This demonstration provides a physical basis for the bifunctional nature of PDIP46. Further work is needed to determine the exact location of the p50 binding region of PDIP46, in order for studies that might lead to generation of mutations that have lost either PCNA or p50 binding.

### Insights from studies of PDIP46 activity using model substrates: a working hypothesis for the mechanism of stimulation of Pol δ4 activity on singly primed ssM13 DNA via PDIP46 facilitation of bypass synthesis through secondary structures

The major and critical outcome of our studies is the discovery that PDIP46 has a profound effect on Pol δ4 activity, which is evident when its effects are examined in assays of primer elongation on singly primed ssM13 DNA. PDIP46 causes an elevation (ca.10-fold) of Pol δ4 activity, which is manifested at low concentrations with an apparent *K*_D_ of ca 34 nM (Figure 5).

**Figure 10:**
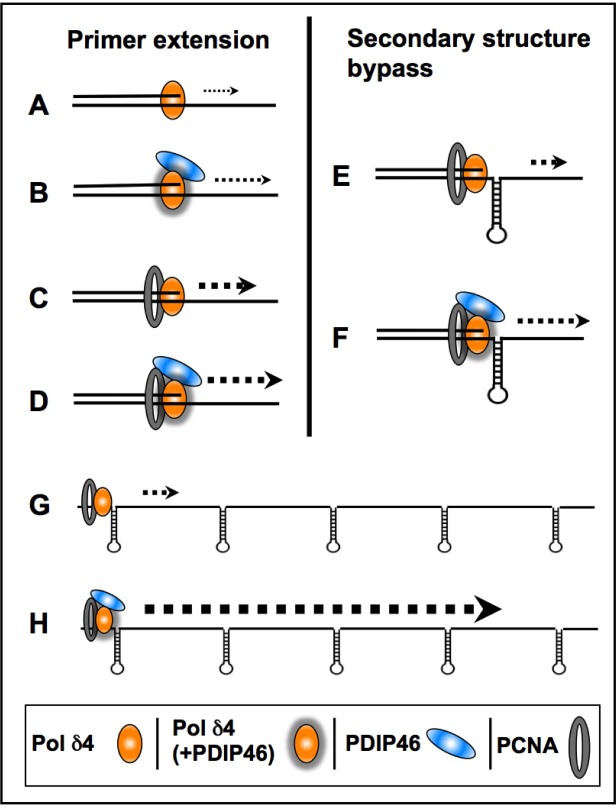
Diagrammatic summary of the effects of PDIP46 on Pol δ4 activity (**A**., **B**.) PDIP46 stimulates Pol δ4 in the absence of PCNA, revealing a direct effect on Pol δ4. (**C**., **D**.) Pol δ4 synthesis in the presence of PCNA. Pol δ4 is strongly stimulated by PCNA alone, due to its conversion to a processive mode of synthesis, but this is further stimulated by PDIP46. (**E**., **F**.) PDIP46 stimulates PCNA-enabled synthesis through a hairpin secondary structure. (This is similar to effects in strand displacement, which are omitted). It is proposed that there is an additive effect of PDIP46 on Pol δ4 synthesis over that observed with oligonucleotides with a single secondary structure; this is illustrated in **G**. and **H**., to show the additive nature of the facilitation of synthesis when multiple stem-loop/hairpin structures are present. This provides a working hypothesis for the potent stimulation of Pol δ4 synthesis of full-length products in the M13 assay by PDIP46. The increases in product formation are qualitatively represented by increases in weight of the dotted arrows for all panels. The direct effects of PDIP46 on Pol δ that indicate alteration in Pol δ function are shown by shadowing of the icons for Pol δ4 (B, D, F).

The experiments using oligonucleotide substrates have provided significant insights into the potential mechanisms that underlie the ability of PDIP46 to stimulate synthesis of full-length products in the M13 assay by Pol δ. A summary of the results of the oligonucleotide experiments is shown diagrammatically in Figure 10. The ability of PDIP46 to affect primer extension in the *absence* of PCNA unequivocally shows that this is mediated via a direct interaction with Pol δ4 (Figure 10A, 10B). In the presence of PCNA, Pol δ4 synthesis is greatly stimulated, but is nevertheless further stimulated by PDIP46 (Figure 10C, 10D). In this instance, we cannot distinguish whether this stimulation is solely due to an effect on Pol δ, or whether it also involves its ability to bind PCNA, as the PDIP46-5A mutation abrogates both p50 and PCNA binding. The detailed mechanism(s) for the ability of PDIP46 to directly stimulate Pol δ remain to be determined by more intensive kinetic studies, such as pre-steady state kinetic analysis, since this could define changes in Pol δ at the catalytic level.

PDIP46 significantly stimulates the ability of Pol δ4 for bypass synthesis through a model hairpin template (Figure 10E, 10F). This provides an explanation of why PDIP46 so strongly facilitates synthesis on the more complex M13 ssDNA template (Figure 10G, 10H). The M13 ssDNA template contains multiple secondary structures, each of which could lead to a slowing of Pol δ4, as well as a potential for causing increased dissociation of Pol δ4. Thus, even modest effects of PDIP46 would be cumulative in the M13 template. The effects of PDIP46, observed over the period required for completion of the synthesis of the 7 kb full-length M13 DNA, would be much greater than those observed with the oligonucleotide substrate containing a single hairpin. This idea also explains why no significant stimulation was observed on the homopolymeric poly(dA) template. In addition, it is an attractive possibility that an interaction of PDIP46 with both PCNA and Pol δ4 could stabilize the Pol δ4/PCNA complex and contribute to the effects of PDIP46 on processive synthesis as well as stimulation of Pol δ4 synthesis through complex templates. Further studies are needed to clarify this issue, and await the development of mutants that can selectively affect PCNA and p50 binding to PDIP46.

It should be noted that while PDIP46 potently stimulates Pol δ4 synthesis on the M13 template, this should also be viewed in the context that even small secondary structures can considerably slow down Pol δ4 synthesis. This also serves as a reminder that analyses of Pol δ4 activity on homopolymeric templates do not reflect those encountered on more complex templates.

The extension of our findings using these substrates to DNA replication in human cells is that PDIP46 could enhance the rate of polymerization by Pol δ4 as well as its ability for bypass synthesis through secondary structures. While it is well accepted that DNA polymerases are impeded by secondary structures, there are few studies of the human DNA polymerases on such templates. Regions of stalling of Pol δ4 have been analyzed in the human FRA16D and FRA3B CSFs (common fragile site) [54, 55], sites of major chromosomal instability [56, 57]. The majority of the pause sites involve relatively small simple hairpins, such as the one used in this study, with loop regions of less than 10 nts and variable stem lengths [54, 55]. The human genome contains significant amounts of sequences that are the recognition sites for replication initiation, transcription, telomeres, amongst others, that pose challenges for human replicative polymerases. These DNA sequences form secondary structures, some as short hairpins and others as complex non-B DNA structures. Replication stalling caused by these sequences is now recognized as a major source of genomic instability that leads to cell death (aging) or transformation (tumorigenesis). These secondary structures can pose impediments to Pol δ, as well as lead to increased error rates [54, 55, 58-60].

### Potential cellular roles of PDIP46 as a modulator of Pol δ4 functions at the replication fork

Our findings have shown that PDIP46 has a profound effect on Pol δ4, while having lesser effects on Pol δ3, particularly in the context of its effect on strand displacement and bypass of hairpin structures. Thus, this establishes a strong basis for a potential role of PDIP46 in replication, specifically in facilitating synthesis through regions of secondary structures. Obviously, there are a large number of cellular helicases that also could be involved in resolving complex structures. Nevertheless, the stimulation of Pol δ4 through secondary structures provides another model for an accessory protein that cumulatively could speed up Pol δ4 synthesis over long stretches of DNA. Further studies of the effects of PDIP46 through more complex secondary structures such as trinucleotide repeats would add to our understanding of its role. Because PDIP46 appears to function by increasing the processivity of Pol δ4, it could act as a partner of Pol δ/PCNA at the replication fork as an “auxiliary” or “accessory” protein. While further research is needed to establish whether PDIP46 is a true accessory protein, and to establish its contributions to DNA replication *in vivo*, our present studies provide a reasonable foundation and rationale for explorations of this possibility. It is relevant that the *in vitro* activity of Pol δ is much slower than the estimated rate of *in vivo* synthesis, so that its function within the replisome might require additional protein factors. Assays of steady state rates of synthesis by Pol δ4 in standard assays give rates of ca. 1 to 3nts/sec [38, 47], while the *in vivo* rate of synthesis in human cells has been estimated to be in the order of 30nts/sec in HeLa cells, as determined by BrdU labeling and DNA fiber analysis [61]. In yeast reconstituted replication systems, the rate of DNA synthesis is also about 10 fold lower than that *in vivo* [36, 62, 63].

There have been extensive studies in yeast by several approaches that support the paradigm for a division of labor for Pol δ and Pol ε at the lagging and leading strands, respectively [36, 64-66], although a very recent study supports a major role for Pol δ in both leading and lagging strands [67]. Less direct experimental evidence is available in mammalian systems as to the designation of the roles of Pol δ and Pol ε. Subcellular localization studies of Pol δ and Pol ε in human cells during cell cycle progression as well as ChIP analyses have provided evidence that Pol δ and Pol ε may operate independently during S phase [68-70]. Our findings that PDIP46 accelerates Pol δ4 synthesis is consistent with a function as an accessory protein that allows Pol δ4 to participate in leading strand synthesis, together with Pol ε.

Our current findings have highlighted the connection between strand displacement and the synthesis through the stem portion of stem-loop secondary structures; the lack of strand displacement activity by Pol δ3 also means that it is less able than Pol δ4 to negotiate stem-loop structures. Thus, we envisage that Pol δ4, with the assistance of PDIP46 may also be utilized in lagging strand synthesis to deal with such structures by switching with Pol δ3. This is consistent with findings that both Pol δ4 and Pol δ3 are proficient in Okazaki fragment processing with Fen1 and DNA ligase I [27]. Our studies would expand the latest model of the replication fork in higher eukaryotes which has Pol δ at both leading and lagging strands [71] to accommodate two forms of Pol δ and the participation of PDIP46.

### PDIP46 as a multifunctional protein and its potential impact on genomic instability

Our studies have now defined PDIP46 as having the potential at the biochemical level to participate in DNA replication. At the same time, PDIP46 has been studied as the SKAR protein whose functions reside in the RRM domain that contains the interaction site for S6K1. SKAR functions provide a linkage to the mTOR pathway for control of cell growth via its recruitment with S6K1 to the exon junction complex where it plays roles in mRNA processing and translational control [29, 30]. SKAR has shown to play a key role in the IFN stimulated expression of genes that are critical for the antileukemic and antineoplastic responses in the use of IFNs in cancer immunotherapy [72]. This involves interferon (IFN)-α induced phosphorylation of SKAR by p90 ribosomal protein S6 kinase (RSK1).

The roles of the C-terminal region may be even more complex, since PDIP46 also interacts with ERH (enhancer of rudimentary homolog) [73], a connection that provides linkages to cell cycle control and DNA replication. ERH is a transcriptional regulator that is required for the splicing of the mitotic motor protein CENP-E, and affects the expression of multiple cell cycle [74] and DNA damage response genes including ATR [75] as well as replication proteins [76].

Another important question that is raised is the potential role PDIP46 has on genomic stability, bearing in mind that PDIP46 has direct effects on Pol δ. As noted previously, these could lead to alterations of the kinetic constants of Pol δ that could affect its intrinsic processivity as well as fidelity, thereby raising the issue of PDIP46 depletion or mutation as a potential source of genomic instability. While the study of PDIP46/SKAR functions are still at an early stage, it is nevertheless apparent that even within its currently known properties that it has the potential to play a role in the cancer process, either in terms of genomic stability directly by affecting DNA replication at the core level of synthesis and fidelity, or in terms of growth regulation and its role in transducing expression of replication and signaling proteins or anti-neoplastic factors as described above. Highly pertinent to the issue of PDIP46/SKAR as a protein that may have potential as a target for oncogenesis are the recent studies that have identified *POLDIP3* as one of a group of genes with altered copy number and expression in metastatic site-derived aggressive cells that exhibited high tumorigenic potential; moreover, reduced *POLDIP3* expression was correlated with decreased overall and relapse free survival in a cohort of 88 patients [77]. Along with this, immunohistochemical tissue staining for PDIP46 in the Human Protein Atlas (http://www.proteinatlas.org) [78] shows a pattern of lowered expression of *POLDIP3* in 20 of the most common cancers. The COSMIC (catalogue of somatic mutations in cancer,) database (http://cancer.sanger.ac.uk/cosmic) [79] also contains data on mutation spectra and altered copy number and expression of *POLDIP3* in cancer tissues. These findings are consistent with a role of PDIP46 in the maintenance of genomic stability.

In summary, our current studies have provided the first analysis of the functional effects of PDIP46 on Pol δ activity. These add to the complexity of PDIP46 functions in relation to those associated with SKAR, but also provide insights and avenues for further dissection of their respective contributions to cellular functions in growth control and genomic stability. Our studies also point to caution in interpretation of the effects of PDIP46/SKAR depletion in cellular studies, because of its bifunctional nature. Thus, future studies in which expression of the two functional domains of PDIP46 in a PDIP46 null cell background are required. Overall, our studies add to the evidence that PDIP46/SKAR is a protein of significant interest in relation to genomic stability and as a potential marker for tumor progression.

## MATERIALS AND METHODS

### Expression and purification of protein reagents

For the pull-down assays, wild type GST-PDIP46, deletion mutants of GST-PDIP46, GST-p50 and truncated GST-p50 fragments were generated by PCR; the PCR generated fragments were digested with restriction endonucleases and subcloned in pGEX-5X-3 (Amersham Pharmacia Biotech,). His-tagged p50 was expressed in the PET33b vector [80]. His-tagged PDIP46 was expressed in the pTacTac vector with eight histidine residues added at its N-terminus. GST fusion or his-tagged proteins were expressed in *E. coli* BL21DE3 (pLysS), and purified by using either glutathione beads (GE Healthcare Life Sciences) or Ni-NTA agarose (Qiagen).

Recombinant human Pol δ4 and Pol δ3 were expressed in insect cells and purified to near homogeneity [47]. PDIP46 was first expressed as the his-tagged-Sumo-PDIP46 fusion protein using the pET Sumo vector (Life Technologies) and purified on Ni-NTA columns. The his-Sumo tags were removed by Sumo protease, and PDIP46 was further purified to near homogeneity by FPLC on Mono S 10/100 ion exchange columns (GE Healthcare Life Sciences). The PDIP46-ΔRRM (deletion of residues 280-351) and PDIP46-5A mutants were generated using QuikChange mutagenesis kits (Stratagene). Concentrations of Pol δ (p125 subunit content), PDIP46 and its mutants were determined by SDS-PAGE with a range of concentrations of catalase as protein standard (Fig. S6, Supplementary Data). Human PCNA was expressed in *E. coli* and purified as previously described [81, 82].

### GST pull-down assays

GST-PDIP46, GST-PDIP46 truncated deletion mutants and GST (control) were incubated with the same amounts of PCNA (or other test proteins) in 600 μl binding buffer (50 mM Tris-HCl, pH 7.8, 1 mM EDTA, 150 mM NaCl, 0.1% NP-40 and 0.2 mM phenylmethylsulfonyl fluoride). The reaction mixtures were incubated by gentle rocking for one hour at 4°C. Packed glutathione beads were added (15 μl) and the suspension further rotated for another hour at the same temperature. The beads were spun down at 2,500 rpm for 5 minutes and washed 8 times with the binding buffer followed by suspension in 1 X SDS loading buffer. The bound proteins were analyzed by SDS-PAGE and Western blotted with antibody against PCNA or other test proteins. Similar protocols were used for other pull-down assays.

### Western blotting, Co-immunoprecipitation and ChIP assays

Western blotting for Pol δ subunits was performed using antibodies against p125, p50, p68 and p12 [12, 14, 19]. PCNA antibodies used were a monoclonal antibody [83, 84] or PC10 (Santa Cruz Biotechnology). A rabbit polyclonal antibody against PDIP46 was generated by Proteintech Group.

A549 human lung adenocarcinoma, HEK 293 and HeLa cells were obtained from ATCC and maintained according to protocols from the supplier [12, 14, 19]. The cells were lysed by sonication and centrifuged at 14,000 rpm for 10 min. Primary antibodies were added overnight followed by addition of A/G agarose beads (Santa Cruz Biotechnology) for 1 hour, at 4° C. The beads were spun down and washed 8 times with RIPA buffer followed by suspension in 2X SDS loading buffer. The bound proteins were analyzed using SDS-PAGE and Western blotted for the test proteins.

ChIP analysis was performed essentially as previously described [14]. A549 cells were grown on 15 cm^2^ plates and cross-linked by addition of formaldehyde (1%) for 10 minutes at room temperature. The cross-linking reaction was terminated by the addition of glycine to a final concentration of 0.25 M. Cells were harvested and ChIP assays using anti-p125 or control IgG were performed using a ChIP-IT Express Enzymatic Kit (Active Motif, Carlsbard, CA). Immunoprecipitated proteins were analyzed by SDS-PAGE and western blotted for PDIP46 and p125. Antibodies to Mcm2 and Ctf4 used as positive controls were obtained from Santa Cruz Biotechnology.

### Native gel electrophoresis

HEK 293 cells from five 75-cm^2^ flasks were harvested and extracts were prepared as described previously [9, 28]. Samples (150 μl, 10 mg/ml protein) were run on 5–15% gradient polyacrylamide gels with a 3.5% stacking gel at 4° C in the absence of SDS. Thyroglobulin (*M*_r_ 669,000), ferritin (*M*_r_ 440,000) and catalase (*M*_r_ 232,000) were used as markers. Gels were run until the migration of the protein standards was limited by pore size (18 h), as established by trial experiments. The proteins were transferred to nitrocellulose membranes and immunoblotted for p125 and PDIP46.

### Assay for processive synthesis by Pol δ on singly primed M13 ssDNA

Assays using singly primed M13 DNA as the template were performed as previously described [12, 47]. Single stranded M13mp18 DNA (7250 bp, New England Biolabs, MA) was primed with a 20-mer oligonucleotide (5′-CTAGAGGATCCCCGGGTACC-3′) complementary to nucleotides 6262–6243 of the M13 genome. The standard reaction contained 40 mM Tris-HCl, pH 7.8, 1 mM dithiothreitol, 0.2 mg/ml BSA, 10 mM MgCl_2_, 0.5 mM ATP, 50mM NaCl, 250 μM each of dTTP, dCTP, and dGTP, and 25 μM cold dATP with 3 μCi of [α-^32^P]-dATP, 2.5 nM primed M13 template, 6 nM human RFC, 6 nM PCNA, 250 nM RPA in a final volume of 16 μl. PCNA was first loaded onto the primed M13 DNA in the presence of RFC, RPA, ATP and Mg^2+^. Pol δ concentrations ranged from 5 nM to 60 nM as indicated in the figure legends. Pol δ was pre-incubated with PDIP46 on ice for 5 min prior to reaction. The reaction was started by the addition of Pol δ, or Pol δ with PDIP46. The reaction mixtures were incubated at 37°C for the indicated times and were terminated by the addition of 120 mM EDTA. The reaction products were run on 1.2% alkaline agarose gels at 70 V for 1.5 h. The gels were dried and visualized using a Molecular Dynamics Storm PhosphorImager system and quantified with ImageQuant software (Amersham Bioscience, NJ).

### Assays for primer extension and strand displacement synthesis using oligonucleotide substrates

The primer/templates used for primer extension and strand displacement were as previously described [27]. The template for primer extension consisted of a 70mer: 5′-biotin-CCT ATC TGA GCA CTA TCA TCG GTC GCA TCG TTG GCT GAA ATC GTG CTG TAG TGG CTG AAT CCC AAC CAA C-3′-biotin, where the ends were tagged with biotin. The 34mer primer sequence was 5′-GTT GGT TGG GAT TCA GCC ACT ACA GCA CGA TTT C-3′. This primer was 5′ end-labeled with ^32^P and annealed to the template [27]. For strand displacement assays, a 31mer blocking oligonucleotide, 5′-ACG ATG CGA CCG ATG ATA GTG CTC AGA TAG G-3′ was also annealed downstream from the primer. Streptavidin was used to block the template ends and PCNA was loaded with RFC [27]. The hairpin substrate consisted of a 64mer template, 5′-GCG ATG CGA CCG ATG ACC CCC CCC TCA TCG GTC GCA TCG CTG GCT GTC AAG GTG CTG TAG TGG C-3′, where the underlined sequences are complementary and formed the hairpin. This was annealed to a 5′ -^32^P-end labeled 19mer primer, 5′-GCC ACT ACA GCA CCT TGA C-3′.

Pol δ and PDIP46 were pre-incubated on ice for 5 min before they were added to the reaction mixture. The reaction contained 50 nM or 100 nM of DNA, Pol δ and PDIP46 as indicated, 50 mM Tris-HCl pH 7.5, 50 mM NaCl, 2 mM dithiothreitol, 0.1mg/ml BSA, 5 mM MgCl_2_, and 0.15 mM dNTP. MgCl_2_ and dNTPs were added to the mixture to start the reactions, and equal volumes of gel loading buffer were added to stop the reactions. The loading buffer contained 50 mM EDTA, 95% formamide, 0.01% bromophenol blue, 0.01% xylene cyanol. Reaction products were subjected to electrophoresis on sequencing gels (16–20% acrylamide/bisacrylamide 19:1 (Bio-Rad), 7.4 M urea, 1 mM EDTA, 90 mM Tris–HCl and 90 mM boric acid). Reaction products were visualized by phosphorimaging with a Molecular Dynamics Storm Phosphorimaging system and quantified with ImageQuant software (Amersham Biosciences).

## SUPPLEMENTARY MATERIAL FIGURES


