# Supplementary material for: PDIP46 (DNA polymerase δ interacting protein 46) is an activating factor for human DNA polymerase δ
Source: Oncotarget. 2016 Jan 27;7(5):6294–313. doi: 10.18632/oncotarget.7034 (PMC4868757; doi:10.18632/oncotarget.7034)
Supplement: Supplementary file 1 [file oncotarget-07-6294-s001.pdf]

PDIP46 (DNA polymerase δ interacting protein 46) is an activating factor for human DNA polymerase δ

Supplementary Material

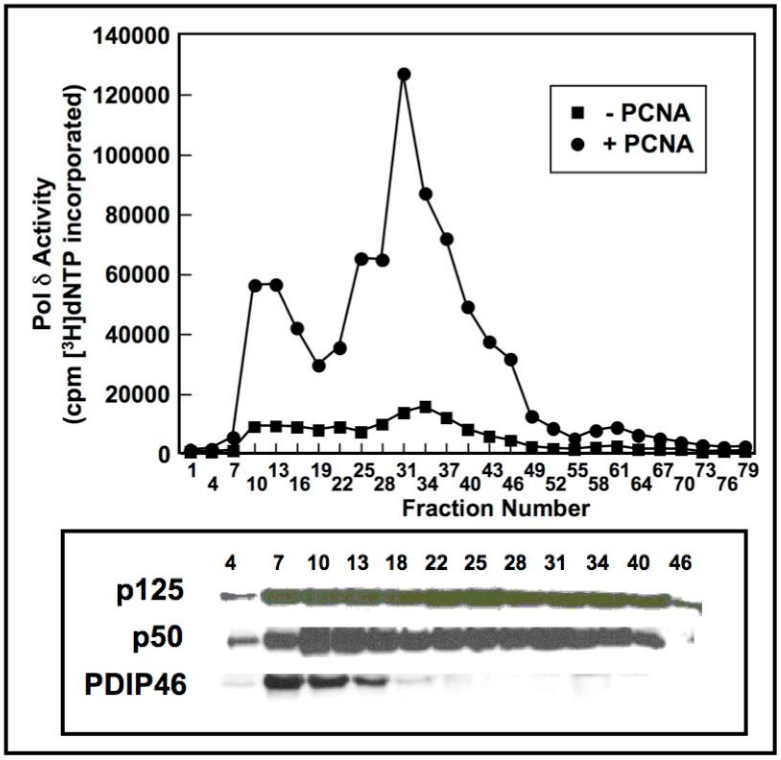

Figure S1. PDIP46 is associated with Pol δ isolated by immunoaffinity chromatography. HeLa cell lysates were subjected to immunoaffinity chromatography on immobilized antibody against the p125 subunit of Pol δ as described previously (12). The upper diagram shows the assay of the eluted fractions for Pol δ activity using poly(dA)/oligo(dT) as the substrate (47). The lower panel shows Western blots of the column fractions for p125, p50 and PDIP46.

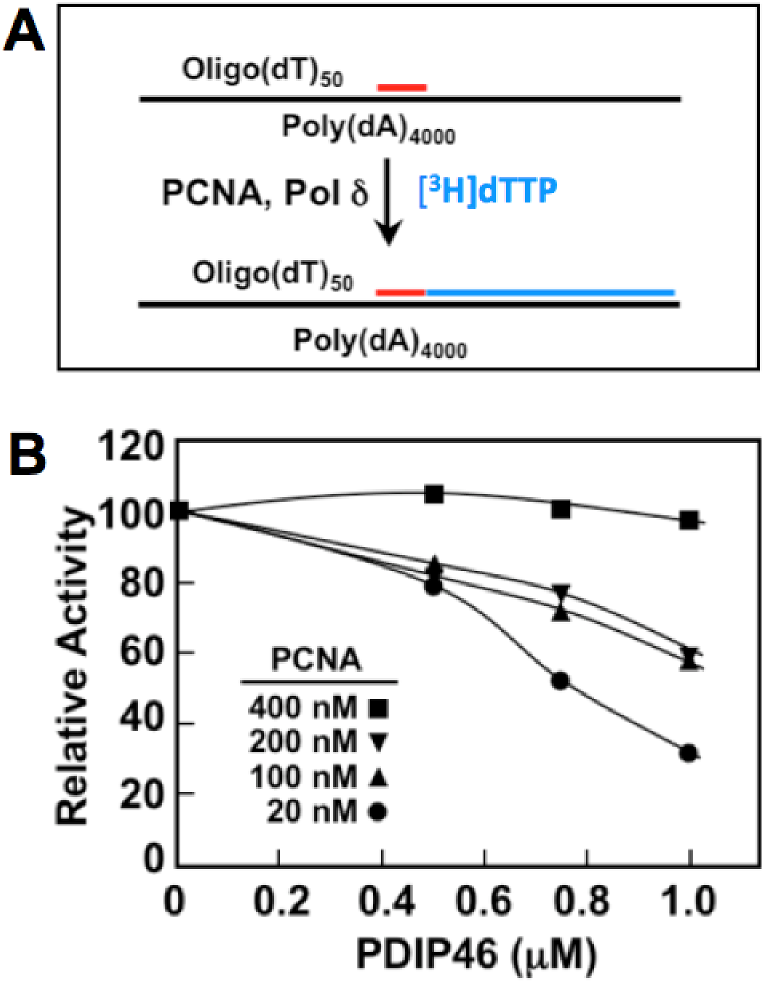

Figure S2. PDIP46 on Pol δ4 in the poly(dA)/oligo(dT) assay. (**A**) Diagrammatic representation of the assay system. The template consists of a linear poly(dA)$_{4000}$ annealed to an oligo(dT)$_{40}$ primer. Note that PCNA is able to access the primer ends so that loading by RFC is not required in the assay. (**B**) Pol δ4 activity was assayed as previously described (47) in the presence of the indicated concentrations of PDIP46. Activities are shown as activities relative to controls in the absence of PDIP46. The assays were performed at PCNA concentrations of 20 nM (circles), 100 nM (triangles), 200 nM (inverted triangles) and 400 nM (squares).

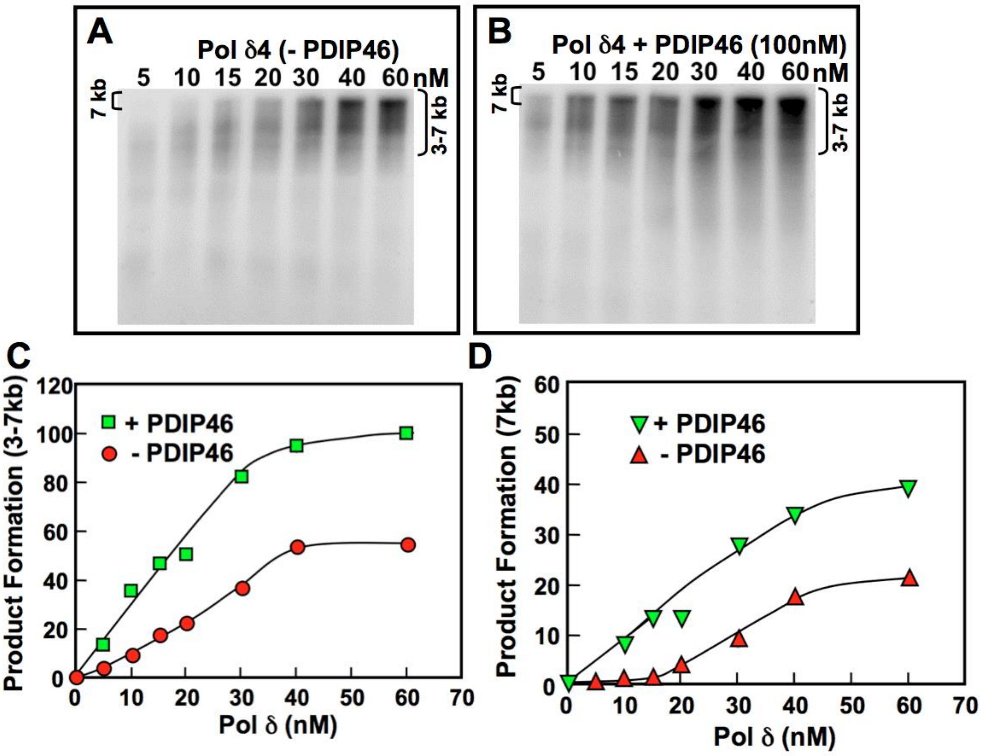

Figure S3. Effects of increasing Pol δ4 concentration at a fixed PDIP46 concentration on product formation in the M13 assay.

(**A, B**) Phosphorimages of product formation by Pol δ4 in the M13 assay at increasing concentrations from 5-60 nM in the absence and presence of 100 nM PDIP46 for 18 min. (**C**) The products in the 3-7 kb range that comprised the majority of the reaction products in the absence (circles) and presence (squares) of PDIP46 from the data in A and B were quantified and plotted against Pol δ4 concentration relative to the maximal value in the presence of 60 nM Pol δ. (**D**) The products in the range of 7 kb in the absence (triangles) and presence (inverted triangles) of PDIP46 from the data in A and B were quantified and plotted against Pol δ4 concentration. *Note the significant lag in formation of full-length products with increasing concentration of Pol δ4 (between 0-20 nM) that disappears in the presence of PDIP46.*

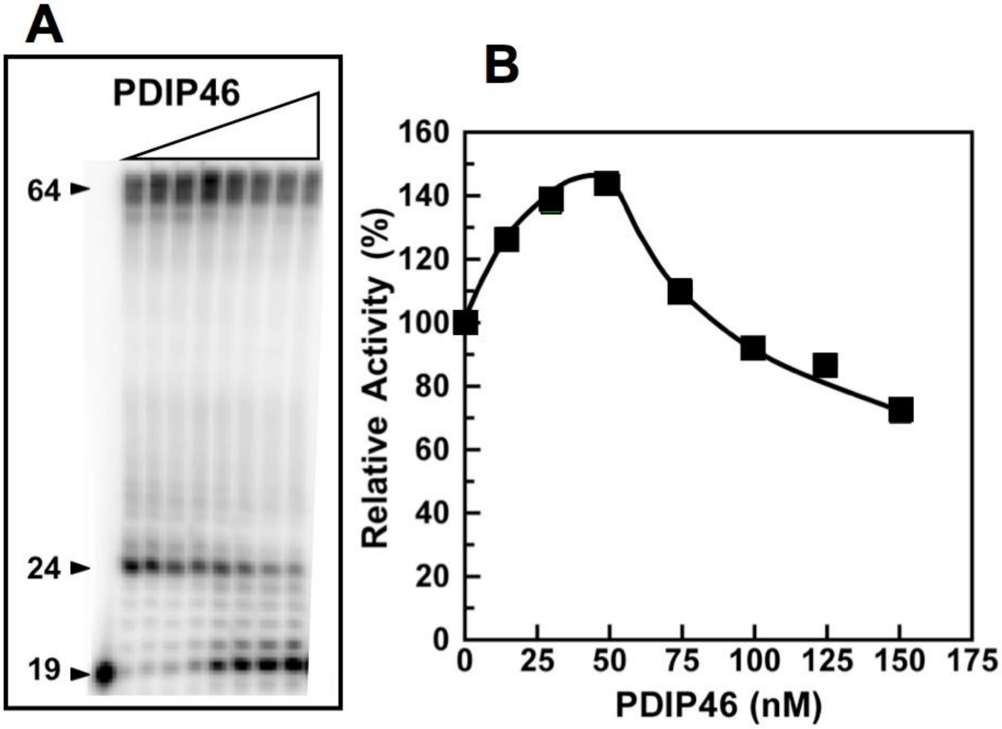

Figure S4. PDIP46 inhibits Pol δ4 synthesis through a hairpin at high concentrations. (**A**) Assays were performed as in Figure 9. Pol δ4 (15 nM) was incubated with increasing concentrations of PDIP46 (0, 15, 30, 50, 70, 100, 125, 150 nM) for 5 min with the hairpin substrate (50 nM) and the reactions were terminated after 8 min. (**B**) The amounts of the 64mer products (as relative product formation with the amounts formed in the absence of PDIP46 taken as 100%) were plotted against PDIP46 concentration.

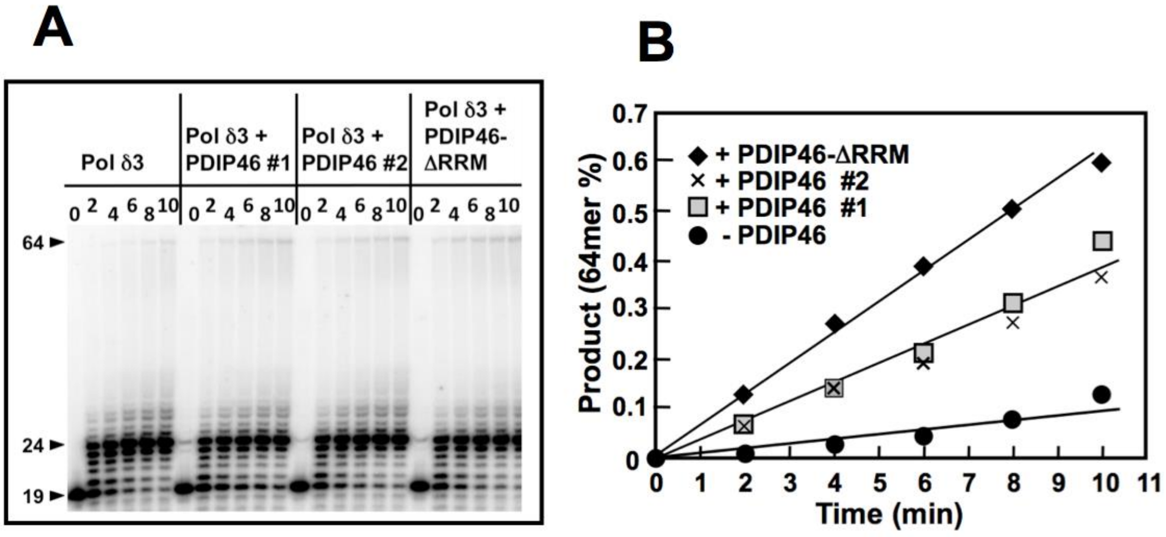

Figure S5. Effects of PDIP46 on Pol δ3 synthesis on a hairpin substrate. **(A)** Pol δ3 (10 nM) was assayed on the hairpin substrate (50 nM) as in Figure 9D in the absence and presence of PDIP46 or its mutants (50 nM) and incubated for the indicated reaction times. Product formation was visualized by phosphorimaging. Two preparations of PDIP46 were used, indicated as PDIP46 #1 and #2. The effects of the mutant proteins in which the RRM was deleted (PDIP46-ΔRRM) were also analyzed. (**B)** The amounts of the 64mer full-length products for A were quantified and plotted as percentage conversion of the primer against time. Data for the control without PDIP46 are shown as solid circles, for PDIP46 #1 as shaded squares, PDIP46 #2 as crosses, PDIP46-ΔRRM as solid diamonds.

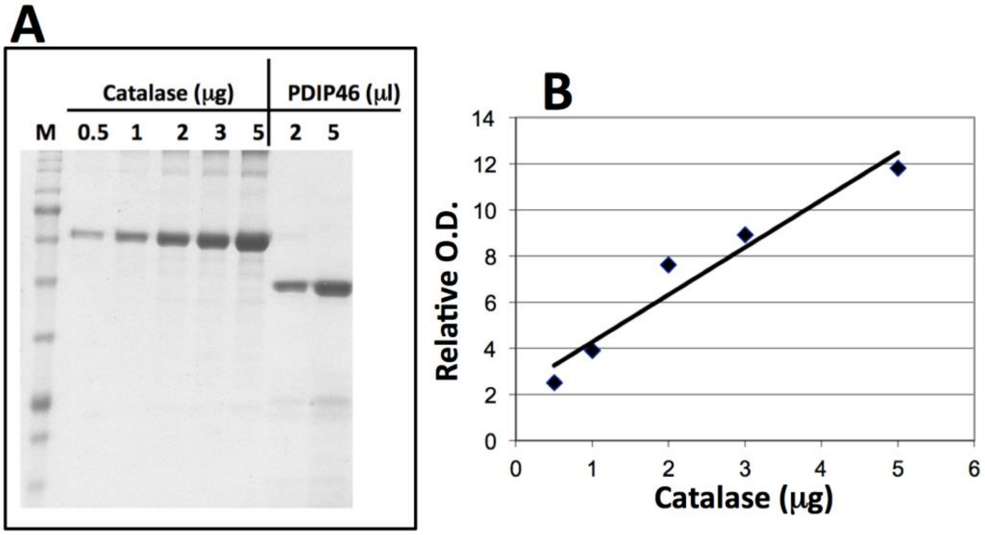

Figure S6. (**A**) SDS-Gel electrophoresis of PDIP46 stained with Coomassie blue. “M” refers to protein molecular weight standards. Catalase in the amounts shown were loaded as indicated. In the right two wells 2 and 5 µl of purified recombinant PDIP46 were loaded. (**B**) The gels were scanned and the intensities of the catalase bands plotted against amounts to obtain a standard curve. The amount of PDIP46 was then determined from the standard curve.
